# Absence of causative genetic association between *Helicobacter pylori* infection and glaucoma: a bidirectional two-sample mendelian randomization study

**DOI:** 10.3389/fgene.2024.1368915

**Published:** 2024-05-24

**Authors:** Yan Zhang, Yihong Huang, Yuyu Wu, Jinying Zhang, Wanzhu Chen, Danfeng Xu, Maosheng Guo

**Affiliations:** ^1^ Department of Ophthalmology, Second Affiliated Hospital of Fujian Medical University, Quanzhou, Fujian, China; ^2^ Department of Neurology, Second Affiliated Hospital of Fujian Medical University, Quanzhou, Fujian, China; ^3^ Department of Ultrasound, Second Affiliated Hospital of Fujian Medical University, Quanzhou, Fujian, China

**Keywords:** *Helicobacter pylori*, mendelian randomization, normal tension glaucoma, primary open-angle glaucoma, pseudo-exfoliation glaucoma

## Abstract

**Background:** While clinical research has indicated a potential link between *Helicobacter pylori* infection and the onset of glaucoma, the causality of this association remains uncertain due to the susceptibility of observational studies to confounding factors and reverse causation.

**Methods:** A comprehensive two-sample bidirectional Mendelian randomization (MR) analysis was conducted to assess the causal connection between *H. pylori* infection and glaucoma. Glaucoma was categorized into primary open-angle glaucoma (POAG), normal tension glaucoma (NTG), and pseudo-exfoliation glaucoma (PEG). Various methods, including inverse variance weighted, MR-Egger regression, weighted median, and mode-based estimator, were employed for effect estimation and pleiotropy testing. To enhance result robustness, a sensitivity analysis was performed by excluding proxy single nucleotide polymorphisms.

**Results:** Genetic predisposition for *H. pylori* infection has no causal effect on glaucoma: (OR 1.00; 95% CI 0.95–1.06, *p* = 0.980), (OR 0.97; 95% CI 0.86–1.09, *p* = 0.550), and (OR 0.99; 95% CI 0.90–1.08, *p* = 0.766) with POAG, NTG, and PEG, respectively. An inverse MR showed no causal effect of POAG, NTG, and PEG on *H. pylori* infection (OR 1.01; 95% CI 0.97–1.05, *p* = 0.693), (OR 1.00; 95% CI 0.98–1.03, *p* = 0.804), and (OR 0.99; 95% CI 0.96–1.01, *p* = 0.363), respectively. Heterogeneity (*p* > 0.05) and pleiotropy (*p* > 0.05) analysis confirmed the robustness of MR results.

**Conclusion:** These results indicated that there was no genetic evidence for a causal link between *H. pylori* and glaucoma, suggesting that the eradication or prevention of *H. pylori* infection might not benefit glaucoma and *vice versa*.

## 1 Introduction

Glaucoma, the second leading cause of blindness globally following cataracts, results in the death of retinal ganglion cells and their axons (Kingman, 2004). It is anticipated that by the year 2040, glaucoma will impact approximately 111.8 million individuals globally, establishing itself as the predominant cause of irreversible vision impairment (Tham et al., 2014). The pathophysiology of glaucoma involves various factors, including infection with *H. pylori*—a widespread pathogen linked to conditions such as Alzheimer’s disease, gastritis, gastric ulcers, and gastric carcinomas (Tsolaki et al., 2012). The investigation into the potential association between *H. pylori* infection and glaucoma dates back to 2001 when Kountouras et al. were among the first to explore this connection (Kountouras et al., 2001). In the subsequent two decades, researchers worldwide have delved into this issue, leading to ongoing controversy surrounding the proposed relationship.

A recent comprehensive review and meta-analysis demonstrated a notable connection between *H. pylori* infection and the overall occurrence of glaucoma, revealing a significant association (OR = 2.08, CI 95% 1.48–2.93) despite a moderate degree of heterogeneity (I2 = 61.54%) (Doulberis et al., 2020). It is important to note that the studies included in this meta-analysis were observational in nature, making it difficult to establish a definitive causal relationship. The question remains whether there is a cause-and-effect link between these two conditions or if they share common predisposing or precipitating factors. To address this uncertainty, a bidirectional two-sample Mendelian randomization (MR) analysis was conducted to clarify the causal relationship between genetically predicted *H. pylori* infection and the occurrence of glaucoma.

Mendelian randomization (MR) analysis has emerged as a powerful tool in epidemiological research for assessing causal relationships between exposures and outcomes. Unlike observational studies, MR analysis utilizes genetic variants, such as single nucleotide polymorphisms (SNPs), as instrumental variables to mimic randomized controlled trials, thereby providing more robust evidence of causality. These genetic variants are randomly allocated at conception and are typically unaffected by environmental confounders, making them ideal instruments for investigating causal associations (Swanson et al., 2017).

Previous studies have explored the causal relationship between dried fruit intake and cardiovascular disease risk, offering insights into dietary interventions for cardiovascular health. Another investigated the bidirectional relationship between gastroesophageal reflux disease and anxiety disorders and depression, unraveling potential pathways linking gastrointestinal health to mental wellbeing. Additionally, research into the roles of the gut microbiome in epilepsy risk has utilized Mendelian randomization to uncover potential mechanisms underlying neurological disorders. These studies exemplify the versatility and applicability of Mendelian randomization in elucidating causal pathways in complex diseases, paving the way for targeted interventions and improved public health strategies (Zeng et al., 2023a; Zeng et al., 2023b; Zeng et al., 2023c).

In our study, we employed a two-sample MR analysis to predict the likelihood of *H. pylori* infection and evaluate its potential association with glaucoma using independent, population-scale GWAS data for glaucoma. Furthermore, we investigated the possibility of reverse causation by considering the incidence of glaucoma as the exposure variable. Our aim was to elucidate the causal relationship between *H. pylori* infection and glaucoma, offering valuable insights for clinical practice.

## 2 Materials and methods

### 2.1 Assumptions and study design of MR

**FIGURE 1:**
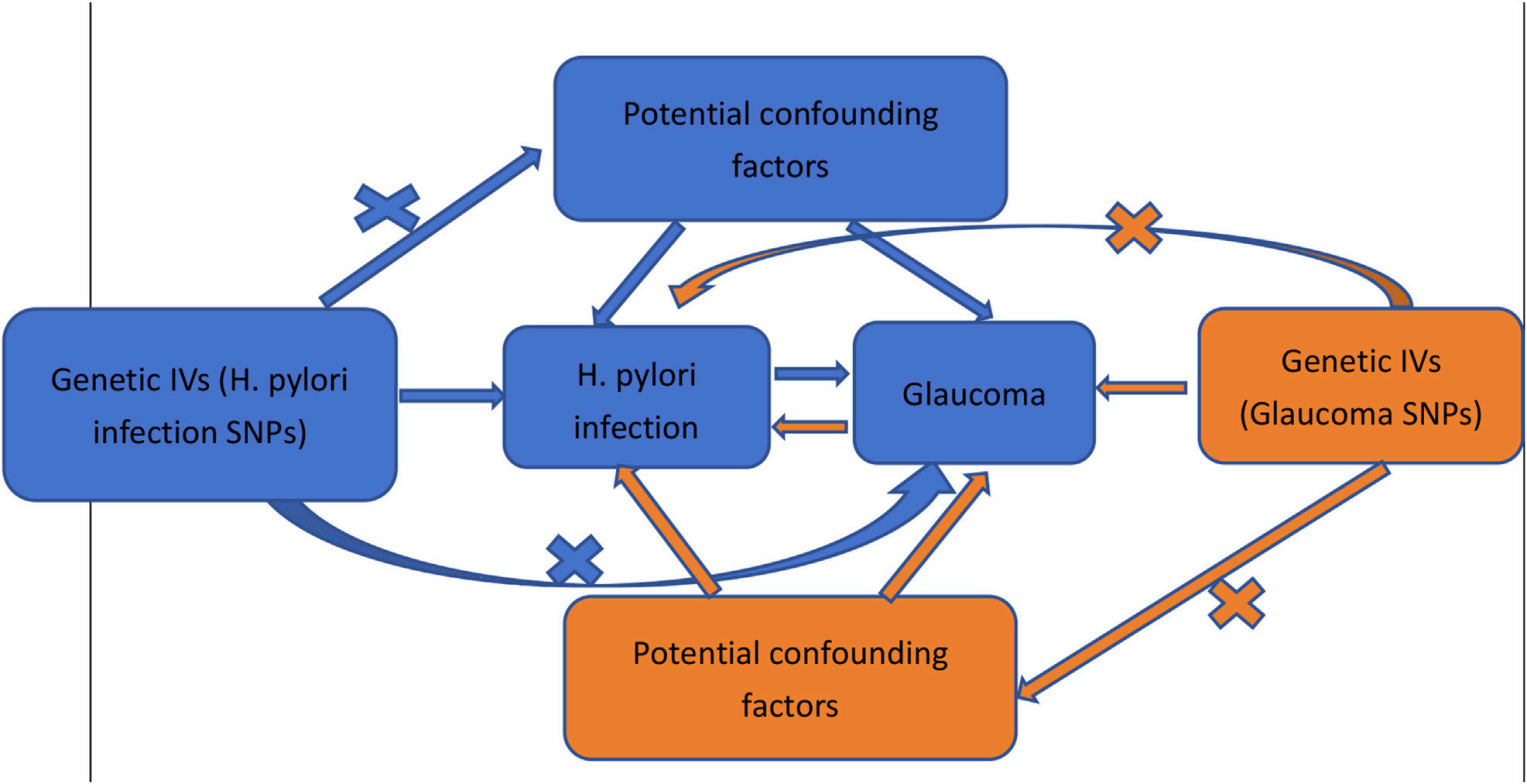
Schematic representation of the bidirectional MR study on the causal relationship between *H. pylori* infection and glaucoma.

The evaluation of causal relationships between glaucoma and *H. pylori* infection was conducted using bidirectional two-sample MR analysis. Summary-level data for *H. pylori* infection and glaucoma were acquired from genome-wide relationship studies (GWAS). In this study, genetic variants were employed as instrument variables (IVs) for the MR analysis. Furthermore, the MR analysis adhered to three core assumptions (Figure 1) to ensure reliable results (Davey Smith and Hemani, 2014): (1) the relevance assumption, indicating strong association of genetic variants with the exposure; (2) the independence assumption, asserting that genetic variants are not linked to any confounders that could mediate pathways from exposure to outcome; and (3) the exclusion-restriction assumption, positing that genetic variants solely influence the outcome through the exposure. Our findings were reported in accordance with the MRSTROBE guidance (Skrivankova et al., 2021). The de-identified public data used in this study for summary-level information is available for download, and ethical approval was obtained from the respective institutions for each GWAS involved in the study.

### 2.2 Data sources description

#### 2.2.1 Data sources

The *H. pylori* infection GWAS summary data were sourced from the publicly available dataset compiled in the European Bioinformatics Institute (EBI) database, accessible at https://gwas.mrcieu.ac.uk/datasets/ieu-b-4905/. Additionally, the GWAS summary statistics for glaucoma, with further divisions into primary open-angle glaucoma (POAG), normal tension glaucoma (NTG), and pseudo-exfoliation glaucoma (PEG) were retrieved from the FinnGen research project database. The specific URLs for the glaucoma data are as follows: https://gwas.mrcieu.ac.uk/datasets/finn-b-H7_GLAUCPRIMOPEN/, https://gwas.mrcieu.ac.uk/datasets/finn-b-H7_GLAUCOMA_NTG/, and https://gwas.mrcieu.ac.uk/datasets/finn-b-H7_GLAUCOMA_XFG/. Detailed information about the GWAS data used in this study can be found in Table 1.

**TABLE 1:** Details of the studies included in the Mendelian randomization analyses.

Phenotype	Consortium	Ethnicity	Sample size	Year	Number of SNPs	Web source
H.pylori	MRC-IEU	European	4,683participants	2021	7,247,045	https://gwas.mrcieu.ac.uk/datasets/ieu-b-4905/
POAG	FinnGenstuy	European	4,433cases and 210,201controls	2021	16,380,455	https://gwas.mrcieu.ac.uk/datasets/finn-b-H7_GLAUCPRIMOPEN/
NTG	FinnGenstuy	European	892cases and 210,201controls	2021	16,380,447	https://gwas.mrcieu.ac.uk/datasets/finn-b-H7_GLAUCOMA_NTG/
PEG	FinnGenstuy	European	1515cases and 210,201controls	2021	16,380,448	https://gwas.mrcieu.ac.uk/datasets/finn-b-H7_GLAUCOMA_XFG/

Note: SNP, single-nucleotide polymorphisms; H.pylor, *Helicobacter pylori*; EBI, european bioinformatics institute; POAG, primary open-angle glaucoma; NTG, normal tension glaucoma; PEG, pseudo-exfoliation glaucoma.

#### 2.2.2 Selection of IVs

To ensure the robustness of the data and the accuracy of the results, a quality check was conducted on the single nucleotide polymorphisms (SNPs) to obtain valid IVs. This involved two criteria: (1) SNPs had to reach the genome-wide significance threshold (*p* < 5 × 10^−8^). Since the number of eligible IVs at this threshold was limited, a more inclusive threshold (*p* < 5 × 10^−5^) was chosen to ensure a more comprehensive result. (2) To satisfy the assumptions of MR, a linkage disequilibrium (LD) analysis was performed using the European-based 1,000 Genome Projects. SNPs failing to meet the criteria (R2 < 0.001, clumping distance = 10,000 kb) were excluded. (3) Palindromic SNPs were also eliminated to mitigate the potential influence of alleles on establishing causality between *H. pylori* infection and glaucoma.

### 2.3 Statistical analysis

Statistical analyses were conducted using R software (Version 4.1.1), employing the “TwoSampleMR” R package for conducting MR analysis to assess the causal link between *H. pylori* infection and glaucoma. A significance level of *p* < 0.05 was utilized to determine the statistical significance, indicating potential evidence for a causal effect.

#### 2.3.1 MR analysis

The Wald ratio (WR) approach was employed to investigate the impact of individual IVs on the causal estimates. In cases without horizontal pleiotropy, the inverse variance weighted (IVW) test served as the primary method for computing unbiased estimates of the causal effects. The choice between a fixed or random effects model for the IVW test was determined by the presence or absence of heterogeneity. The effect size was expressed through OR and their corresponding 95% confidence intervals (CI). Additional methods for MR analysis included the (Weighted Median) WM method (Bowden et al., 2016) and the MR-Egger test (Bowden et al., 2015). WM results were considered significant causal effect values when the number of SNPs exhibiting heterogeneity surpassed 50%, while the MR-Egger results remained valid when SNPs with pleiotropy exceeded 50%.

#### 2.3.2 Sensitivity analysis

The heterogeneity was examined using the Cochrane’s Q test, and variables with a *p* < 0.05 were regarded as heterogeneous. The MR-Egger regression’s intercept was employed to evaluate potential pleiotropy in instrumental variables, with horizontal pleiotropy considered absent if *p* > 0.05. To enhance data robustness, the leave-one-out method was also employed for validation.

## 3 Results

To conduct the MR analysis, a broader significance threshold (*p* < 5 × 10^−5^) was chosen due to zero number of eligible IVs (*p* < 5 × 10^−8^). Significant and independent SNPs were included, while those with an F-statistics <10 were excluded (F statistics = R2 (*n* − k − 1)/k (1 − R2), where R2 refers to variance of exposure explained by selected IVs, n refers to the sample size, and k refers to the number of IVs).Ultimately, a total of 66, 110, 68, and 60 SNPs related to *H. pylori* infection, POAG, NTG, and PEG were selected for the two-sample MR analysis.

**FIGURE 2:**
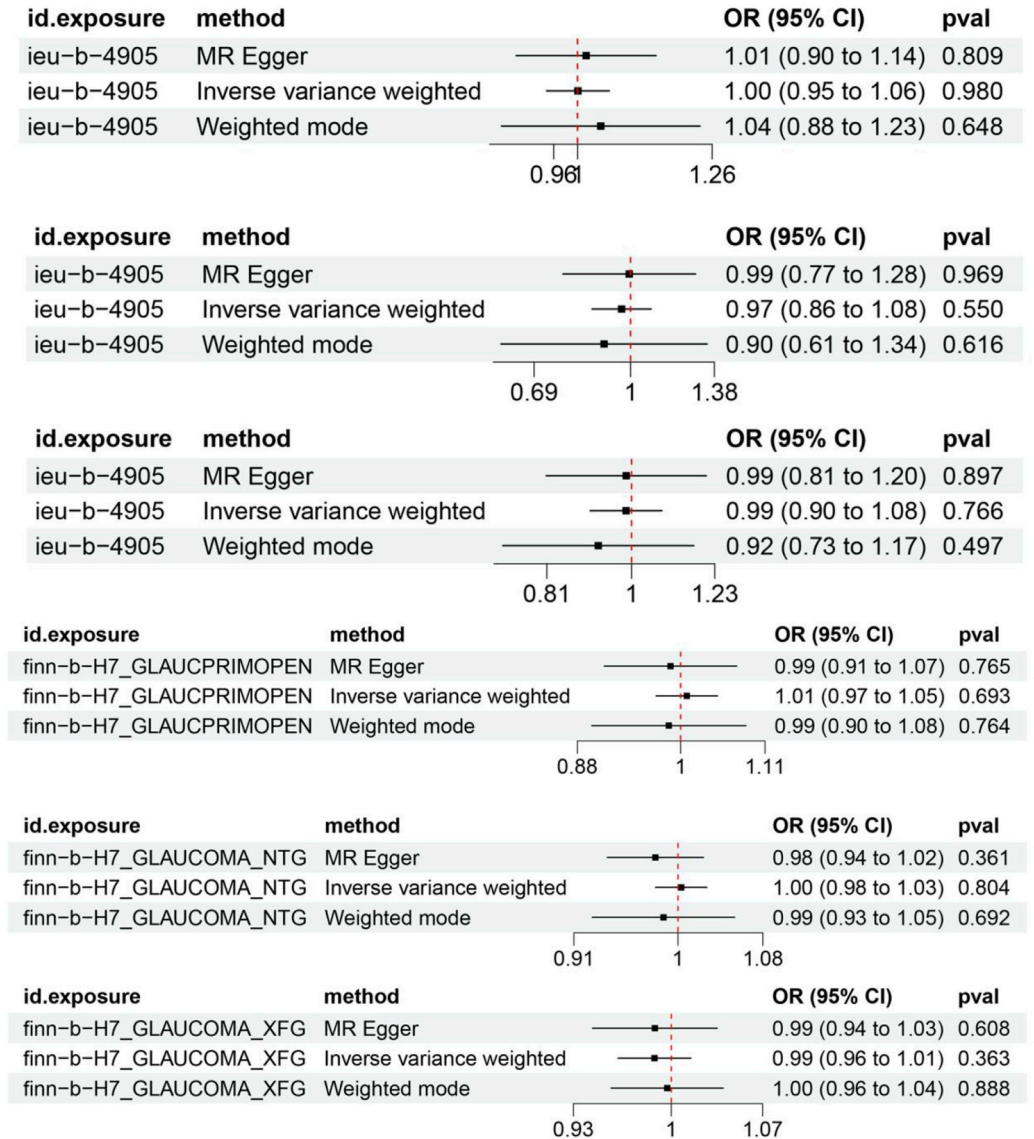
Forest plot of Mendelian randomization results of *H. pylori* effect on POAG, NTG, and PEG. ieu-b-4905 is the *H. pylori* infection GWAS summary data, finn-b-H7_GLAUCPRIMOPEN is the primary open-angle glaucoma GWAS summary data, finn-b-H7_GLAUCOMA_NTG is the normal tension glaucoma GWAS summary data, finn-b-H7_GLAUCOMA_XFG is the pseudo-exfoliation glaucoma GWAS summary data.

**FIGURE 3:**
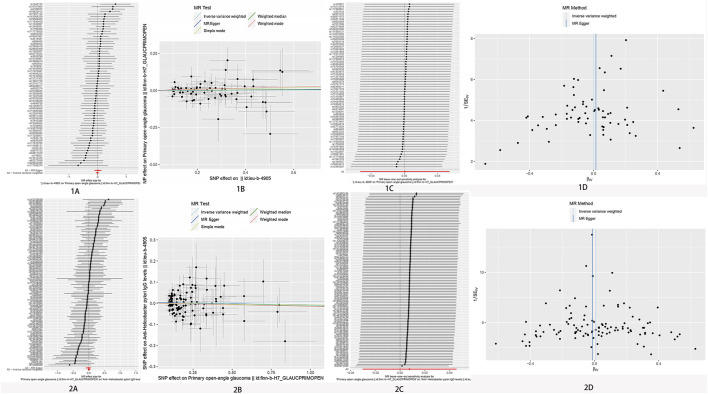
No significant causal relationship between *H. pylori* infection and POAG risk. **(A)** Forest plot where each black point represents the log odds ratio (OR) and red points showing the combined causal estimate using all SNPs together in a single instrument, using each of the two different methods (inverse variance weighted [IVW] random effects and MR-Egger). Horizontal lines denote 95% confidence intervals (95%CIs). **(B)** Scatter plot of the causal relationships between *H. pylori* infection and POAG using different MR methods. Slope of each line corresponding to the causal estimates for each method. Individual SNP effect on the outcome (point and vertical line) against its effect on the exposure (point and horizontal line) is delineated in the background. **(C)** MR leave-one-out sensitivity analysis. Each black point represents the IVW MR method applied to estimate the causal effect excluding that particular variant from the analysis. The red point depicts the IVW estimate using all SNPs. There are no instances where the exclusion of one particular SNP leads to dramatic changes in the overall result. **(D)** Funnel plot showing the causal effect estimated using each individual SNP as a separate instrument against the inverse of the standard error of the causal estimate. Vertical lines show the causal estimates using all SNPs combined into a single instrument for each of the two different methods (IVW random effects and MR-Egger). There is no significant asymmetry in the plot. 1. Causal estimates for *H. pylori* infection on POAG. 2. Causal estimates for POAG on *H. pylori* infection.

**FIGURE 4:**
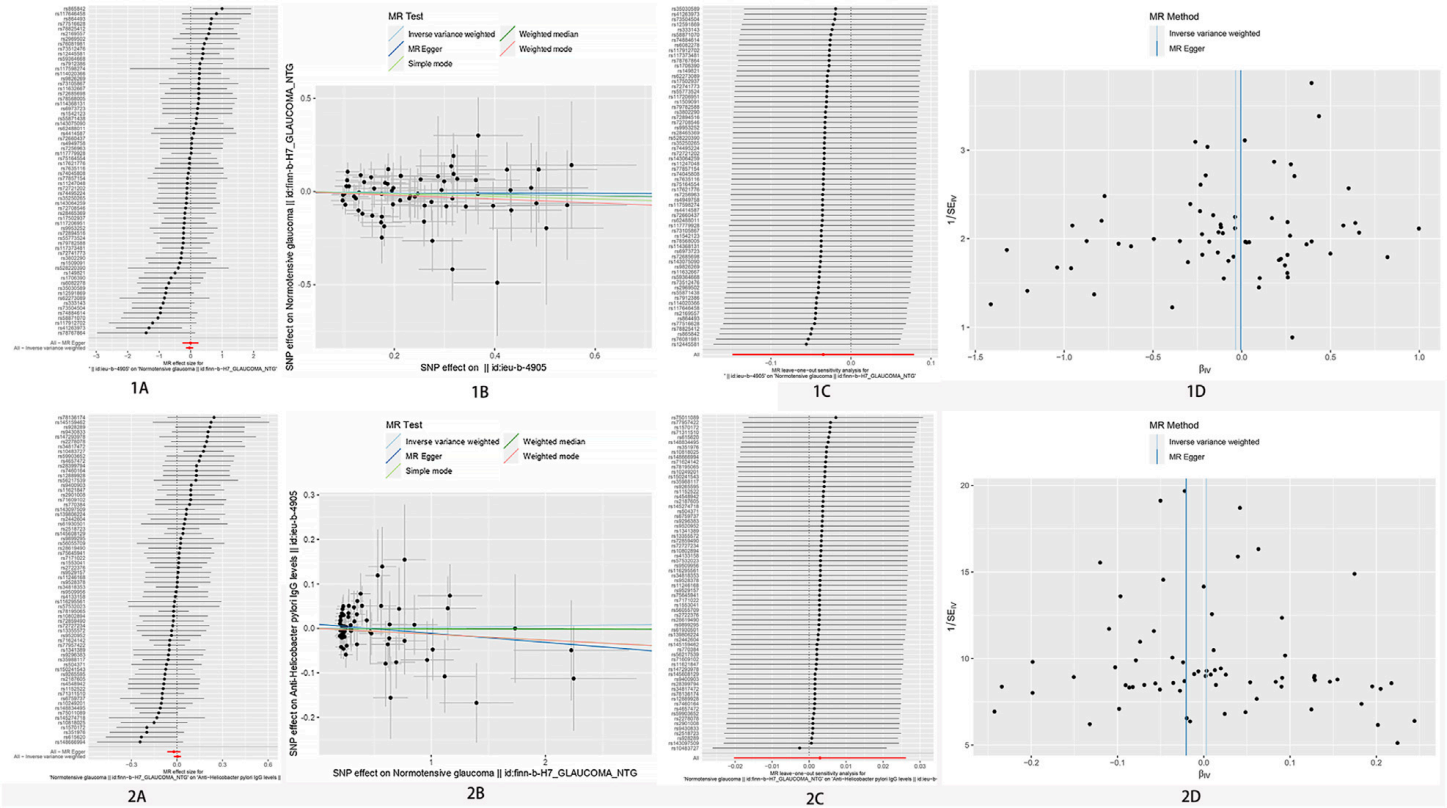
No significant causal relationship between *H. pylori* infection and NTG risk. **(A)** Forest plot where each black point represents the log odds ratio (OR) and red points showing the combined causal estimate using all SNPs together in a single instrument, using each of the two different methods (inverse variance weighted [IVW] random effects and MR-Egger). Horizontal lines denote 95% confidence intervals (95%CIs). **(B)** Scatter plot of the causal relationships between *H. pylori* infection and POAG using different MR methods. Slope of each line corresponding to the causal estimates for each method. Individual SNP effect on the outcome (point and vertical line) against its effect on the exposure (point and horizontal line) is delineated in the background. **(C)** MR leave-one-out sensitivity analysis. Each black point represents the IVW MR method applied to estimate the causal effect excluding that particular variant from the analysis. The red point depicts the IVW estimate using all SNPs. There are no instances where the exclusion of one particular SNP leads to dramatic changes in the overall result. **(D)** Funnel plot showing the causal effect estimated using each individual SNP as a separate instrument against the inverse of the standard error of the causal estimate. Vertical lines show the causal estimates using all SNPs combined into a single instrument for each of the two different methods (IVW random effects and MR-Egger). There is no significant asymmetry in the plot. 1. Causal estimates for *H. pylori* infection on NTG. 2. Causal estimates for NTG *on H. pylori* infection.

**FIGURE 5:**
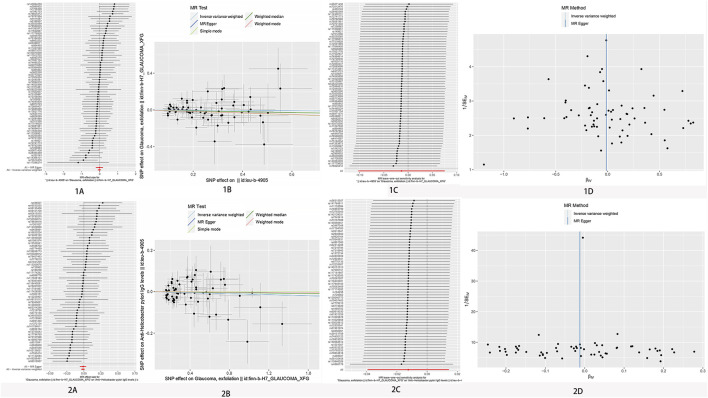
No significant causal relationship between *H. pylori* infection and PEG risk. **(A)** Forest plot where each black point represents the log odds ratio (OR) and red points showing the combined causal estimate using all SNPs together in a single instrument, using each of the two different methods (inverse variance weighted [IVW] random effects and MR-Egger). Horizontal lines denote 95% confidence intervals (95%CIs). **(B)** Scatter plot of the causal relationships between *H. pylori* infection and POAG using different MR methods. Slope of each line corresponding to the causal estimates for each method. Individual SNP-effect on the outcome (point and vertical line) against its effect on the exposure (point and horizontal line) is delineated in the background. **(C)** MR leave-one-out sensitivity analysis. Each black point represents the IVW MR method applied to estimate the causal effect excluding that particular variant from the analysis. The red point depicts the IVW estimate using all SNPs. There are no instances where the exclusion of one particular SNP leads to dramatic changes in the overall result. **(D)** Funnel plot showing the causal effect estimated using each individual SNP as a separate instrument against the inverse of the standard error of the causal estimate. Vertical lines show the causal estimates using all SNPs combined into a single instrument for each of the two different methods (IVW random effects and MR-Egger). There is no significant asymmetry in the plot. 1. Causal estimates for *H. pylori* infection on PEG. 2. Causal estimates for PEG on *H. pylori* infection.

An IVW model incorporating random effects produced a combined MR estimate indicating that there is no evidence to substantiate a causal link with *H. pylori*: (OR 1.00; 95% CI 0.95–1.06, *p* = 0.980), (OR 0.97; 95% CI 0.86–1.09, *p* = 0.550), and (OR 0.99; 95% CI 0.90–1.08, *p* = 0.766) with POAG, NTG, and PEG, respectively (Figure 2; Table 2). An inverse MR showed no causal effect of POAG, NTG, and PEG on *H. pylori* infection (OR 1.01; 95% CI 0.97–1.05, *p* = 0.693), (OR 1.00; 95% CI 0.98–1.03, *p* = 0.804), and (OR 0.99; 95% CI 0.96–1.01, *p* = 0.363), respectively (Figures 3–5). The outcomes from assessing candidate SNPs using weighted median and weighted mode estimates closely resembled those obtained from the random-effects IVW model. No indications of heterogeneity were observed in the IVW and MR-Egger analyses (*p* > 0.05). Additionally, no evidence of directional horizontal pleiotropy was identified in the analysis, as evidenced by the MR-Egger intercept test (*p* > 0.05). Further scrutiny through leave-one-out analysis, MRPRESSO, and funnel plot analysis failed to identify any significant outliers.

**TABLE 2:** Forest plot of Mendelian randomization results of HP effect on POAG, NTG, and PEG.

Exposure	Outcome	N. SNPs	IVW		Pval	Heterogeneity		Pleiotropy
			OR	95%CI		MR Egger	IVW	
*H. pylori*	POAG	66	1.00	0.96 to 1.06	0.980	>0.05	>0.05	>0.05
	NTG	66	0.97	0.86 to 1.08	0.550	>0.05	>0.05	>0.05
	PEG	66	0.99	0.99 to 1.08	0.766	>0.05	>0.05	>0.05
POAG	*H. pylori*	110	1.01	0.97 to 1.05	0.693	>0.05	>0.05	>0.05
NTG		68	1.00	0.96 to 1.03	0.804	>0.05	>0.05	>0.05
PEG		60	0.99	0.96 to 1.01	0.363	>0.05	>0.05	>0.05

Note: OR, odds ratio; Heterogeneity, *p*-value for Cochran’s Q test; Pleiotropy, *p*-value for MR-Egger intercept test; N. SNPs, number of single-nucleotide polymorphisms used in MR; POAG, primary open-angle glaucoma; NTG, normal tension glaucoma; PEG, pseudo-exfoliation glaucoma; *H. pylori*, *Helicobacter pylori*.

## 4 Discussion

In the present study, we investigated the association between glaucoma risk and *H. pylori* infection using the bidirectional two-sample MR method, firstly, and demonstrating that there was no significant causal relationship between *H. pylori* infection and the risk of glaucoma.

In the early 2000 s, studies on the association between glaucoma and *H. pylori* marked a significant development. In 2001, an investigation on the potential link between *H. pylori* infection and glaucoma was undertaken by Kountouras et al. (2001) who were among the pioneers in this exploration. They postulated that a theoretical relationship exists between glaucoma and *H. pylori* infection, based on the following considerations: (1) both ailments predominantly affect older adults in the developed world; (2) chronic *H. pylori* infection has the potential to induce systemic disorders affecting vascular tone through the release of vasoactive and proinflammatory substances; and (3) *H. pylori* infection correlates with increased platelet activation and aggregation induced by arteriosclerosis. To investigate this hypothesis, a prospective non-randomized comparative study was conducted, (based on a sample of 32 patients with POAG, 9 patients with PEG and a control group of 30 age-matched anemic patients). The objective was to ascertain the prevalence of *H. pylori* infection in both patients with glaucoma and the anemic control participants. The findings of the study led to the conclusion that *H. pylori* infection is encountered more frequently among patients with glaucoma compared to the general population. These results were presented in part at the sixth congress of the European Glaucoma Society in London, England, in June 2000.

In a continuation of this investigation (Kountouras et al., 2002), the same researchers demonstrated that over an extended period, individuals with glaucoma experience advantages in terms of the regulation of intraocular pressure and visual field parameters when *H. pylori* is effectively eliminated. The same research team further identified the presence of positive IgG antibodies of *H. pylori* in the anterior chamber of the eyes of patients with POAG and PEG, as opposed to the control group (Kountouras et al., 2003). In a 2011 study by Kountouras and Zavos (Zavos et al., 2012), the histological examination of eye biopsies from patients with POAG revealed the presence of *H. pylori* bacteria. Conversely, the results of a Canadian prospective case-control study suggested no association between exposure to *H. pylori* infection and open-angle glaucoma (Galloway et al., 2003). Furthermore, in an Israeli prospective, population-based study (Kurtz et al., 2008), it was concluded that neither *H. pylori* infection nor seropositivity for virulent CagA-bearing *H. pylori* strains is significantly associated with the occurrence of any type of glaucoma.

Interestingly, Chen et al. conducted a cohort study in Taiwan involving 6,061 patients with peptic ulcer undergoing *H. pylori* eradication therapy (Chen et al., 2015) The findings revealed that irrespective of whether *H. pylori* eradication was initiated early or late, it did not significantly decrease the risk of glaucoma in individuals with peptic ulcer disease when compared to a normal control group. The existing body of research examining the potential connection between *H. pylori* infection and glaucoma has produced conflicting results, with some studies suggesting a positive association and others finding no clear link, leading to ongoing controversy in this area.

Hence, Doulberis et al. undertook a comprehensive examination and synthesis of observational studies (Doulberis et al., 2020). The meta-analysis comprised 15 studies involving 2,664 participants (872 with glaucoma and 1,792 controls). The findings revealed a positive correlation between active *H. pylori* infection and glaucoma. However, when considering glaucoma subtypes, the association was significant for *H. pylori* infection with POAG or NTG, but not with PEG.

While many observational studies have explored potential risk factors for glaucoma, the causal link between these factors and the onset of glaucoma remains uncertain due to the constraints of observational research. In contrast, MR serves as an epidemiological analytical approach that enhances causal inference. It mimics a naturally occurring randomized controlled trial, providing a valuable tool for identifying risk factors and causal relationships with fewer confounding factors compared to traditional observational epidemiological studies (Smith, 2010; Birney, 2022).

The findings of the present study contradict those of the meta-analysis, indicating that an *H. pylori* infection does not pose a risk for glaucoma. Consequently, it is reasonable to consider the possibility that these conditions may have shared predisposing or precipitating factors. Various theories explaining the pathogenic mechanism of this condition have been suggested, given that both illnesses are more prevalent among older adults (Eusebi et al., 2014; Kang and Tanna, 2021).

One potential theory suggests that aging may be a shared factor in both *H. pylori* infection and glaucoma (Doulberis et al., 2020). Given these factors, additional research is necessary to thoroughly explore the potential influence of *H. pylori* infection on the development of glaucoma associated with aging.

Nevertheless, our study has certain constraints. Initially, the identification of *H. pylori* infection relied on serological testing within the GWAS data, introducing potential bias in the detection of *H. pylori* infection. Additionally, our dataset exclusively comprised the European population, implying that the applicability of our findings to non-Europeans should be approached with caution. Lastly, we incorporated only those SNPs with a genome-wide significance level (*p* < 5 × 10^−5^), excluding variants genuinely associated with the condition that did not meet this stringent *p*-value threshold, and the sample size for *H. Pylori* may suffer from having a small sample size of less than 5,000 participants.

## 5 Conclusion

Our findings suggest that there is insufficient genetic evidence to support a causal relationship between *H. pylori* infection and glaucoma. Therefore, the eradication or prevention of *H. pylori* infection may not have a significant impact on the development or progression of glaucoma, and *vice versa*. These results underscore the complexity of the relationship between *H. pylori* and glaucoma and emphasize the need for further research to elucidate any potential causal mechanisms or interactions between these conditions.

## Data Availability

The original contributions presented in the study are included in the article/Supplementary Material, further inquiries can be directed to the corresponding author.

## Abbreviations

*H. pylori*, *Helicobacter pylori*; POAG, primary open-angle glaucoma; NTG, normal tension glaucoma; PEG, pseudo-exfoliation glaucoma; IVW, inverse variance weighted; SNPs, single nucleotide polymorphisms; MR, Mendelian randomization; GWAS, genome-wide relationship studies; EBI, European Bioinformatics Institute; CI, confidence interval; WM, weighted median; WR, Wald ratio; OR, Odds Ratio; MR-PRESSO, Mendelian Randomization Pleiotropy RESidual Sum and Outlier.
