# Supplementary material for: Absence of causative genetic association between Helicobacter pylori infection and glaucoma: a bidirectional two-sample mendelian randomization study
Source: Front Genet. 2024 May 24;15:1368915. doi: 10.3389/fgene.2024.1368915 (PMC11157063; doi:10.3389/fgene.2024.1368915)
Supplement: Supplementary file 1 [file DataSheet1.docx]

**Table S1 Specific information about IVs.**

| H. pylori against POAG | 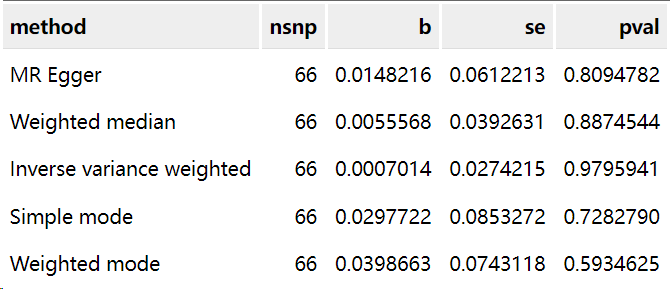 |
| --- | --- |
| H. pylori against NTG | 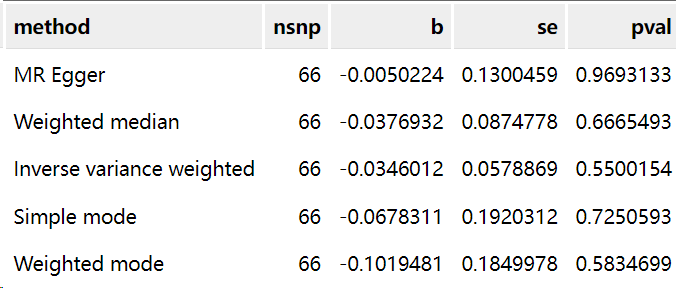 |
| H. Pylori against PEG | 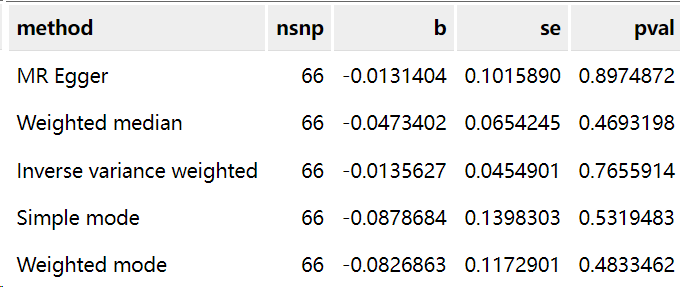 |
| POAG against H. pylori | 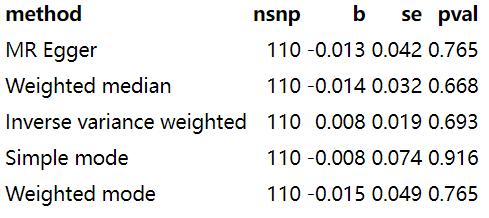 |
| NTG against H. pylori | 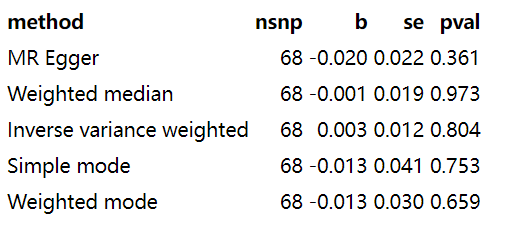 |
| PEG against H. pylori | 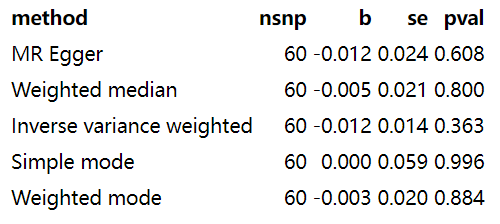 |
| POAG: primary open-angle glaucoma;  NTG: normal tension glaucoma;  PEG: pseudo-exfoliation glaucoma;  H. Pylori: Helicobacter pylori | |
